# A random forest model based classification scheme for neonatal amplitude-integrated EEG

**DOI:** 10.1186/1475-925X-13-S2-S4

**Published:** 2014-12-11

**Authors:** Weiting Chen, Yu Wang, Guitao Cao, Guoqiang Chen, Qiufang Gu

**Affiliations:** 1 Software Engineering Institute, East China Normal University, 3663 North Zhongshan Road, 200062, Shanghai, China; 2 Shanghai Children's Hospital of Fudan University, 399 Wanyuan Road, 201102, Shanghai, China

## Abstract

**Background:**

Modern medical advances have greatly increased the survival rate of infants, while they remain in the higher risk group for neurological problems later in life. For the infants with encephalopathy or seizures, identification of the extent of brain injury is clinically challenging. Continuous amplitude-integrated electroencephalography (aEEG) monitoring offers a possibility to directly monitor the brain functional state of the newborns over hours, and has seen an increasing application in neonatal intensive care units (NICUs).

**Methods:**

This paper presents a novel combined feature set of aEEG and applies random forest (RF) method to classify aEEG tracings. To that end, a series of experiments were conducted on 282 aEEG tracing cases (209 normal and 73 abnormal ones). Basic features, statistic features and segmentation features were extracted from both the tracing as a whole and the segmented recordings, and then form a combined feature set. All the features were sent to a classifier afterwards. The significance of feature, the data segmentation, the optimization of RF parameters, and the problem of imbalanced datasets were examined through experiments. Experiments were also done to evaluate the performance of RF on aEEG signal classifying, compared with several other widely used classifiers including SVM-Linear, SVM-RBF, ANN, Decision Tree (DT), Logistic Regression(LR), ML, and LDA.

**Results:**

The combined feature set can better characterize aEEG signals, compared with basic features, statistic features and segmentation features respectively. With the combined feature set, the proposed RF-based aEEG classification system achieved a correct rate of 92.52% and a high F1-score of 95.26%. Among all of the seven classifiers examined in our work, the RF method got the highest correct rate, sensitivity, specificity, and *F*_1_-score, which means that RF outperforms all of the other classifiers considered here. The results show that the proposed RF-based aEEG classification system with the combined feature set is efficient and helpful to better detect the brain disorders in newborns.

## Background

Over the past decades, modern medical advances have greatly increased the survival rate of term and preterm infants [1]. Based on modern medical research, brain permanent damage can be minimized before it becomes irreversible [2]. Amplitude-integrated electroencephalography is an important tool for the neurological assessment of critically ill newborns [3]. Compared with imaging techniques such as Magnetic Resonance Imaging (MRI), aEEG is more suitable to continuously monitor the brain activity, which could record tracking changes and the maturation process of brain. Benefiting from the non-intrusive nature and high availability of aEEG, it is easy to be applied to portable bedside equipment.

The cerebral function monitor (CFM) was created in the 1960s by Douglas Maynard and first applied clinically by Pamela Prior [4]. In 1970s and early 1980s, Ingmar Rosén and Nils Svenningsen introduced the CFM in the intensive monitoring of brain function in newborns [5][6]. Later, Lena Hellström-Westas started to evaluate the method in the neonatal intensive care unit (NICU) [7].

AEEG signal is derived from a reduced EEG which can be captured by CFM. Unlike the standard EEG, whose setting up and interpreting are labor intensive, aEEG signals are recorded from limited channels with symmetric parietal electrodess [8]. The aEEG processing scenario includes an asymmetric band pass filter with pass band of 2-15Hz, semi-logarithmic amplitude compression and time compression. The filtering will minimize artifacts from sweating, movements, muscle activity and electrical interference. The amplitude is semilogarithmic amplitude compression (linear display 0-10 *µV *; logarithmic display 10-100 *µV*). Continuous aEEG monitoring offers a possibility to directly monitor the functional state of the brain over hours and days. Toet et al.[9] gave a comparison between amplitude integrated electroencephalogram and standard electroencephalogram in neonates and pointed out CFM is a reliable tool for monitoring background patterns (especially normal and severely abnormal ones). Brain monitoring with aEEG is also reported to can better define brain injury and predict out-come than many other methods [3,10].

**Figure 1:**
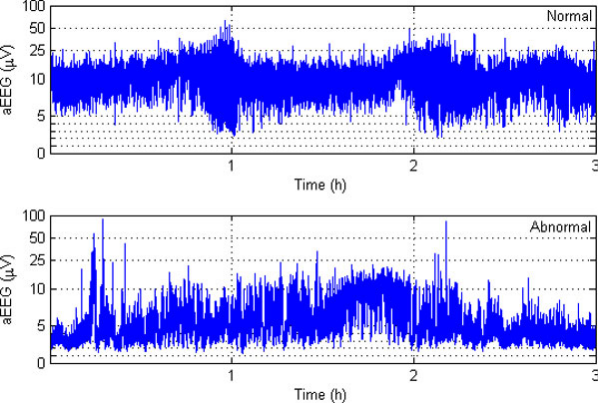
**Two typical aEEG background activities**.

AEEG tracings are described and classified in several different ways, depending on whether normal or abnormal circumstances are evaluated and whether term or preterm infants are studied [9]. A number of publications have described the normal development of aEEG patterns in full-term and preterm infants [10][11][12]. Figure 1 illustrates two typical background activities of normal and abnormal aEEG traces. Clinical aEEG monitoring can reveal abnormal brain activities, but there is a potential possibility that the abnormality would pass unrecognized by users [8]. A study on an automatic method for detecting the cerebral activity based on aEEG can be helpful to avoid such unrecognition. In [13], a statistical distribution feature of aEEG signal was proposed. In [14], an algorithm for the automatic detection of seizures in aEEG was proposed, based on a sudden increase of the aEEG lower boundary which is the characteristic change caused by electrographic neonatal seizures.

To apply machine learning algorithms to aEEG interpretation task, the problem can be considered as a classification problem of signal. Different machine learning algorithms have been used for classification tasks. Among them, Random Forest (RF) and Support Vector Machines (SVMs) are two widely used algorithms. Some studies reported that RF performed better in classification tasks for complex data [11]. In the previous work presented in the 2013 IEEE International Conference on Bioinformatics and Biomedicine (BIBM) [12], we explored a random forest model with combined features for aEEG classification. The experiment results showed that RF achieved better performance than other machine learning algorithms, indicating it is a promising algorithm for the automatic aEEG signal interpretation. This paper is an extension to our previous work, focusing on the optimizing the configuration of the classification scheme.

## Methods

**Figure 2:**
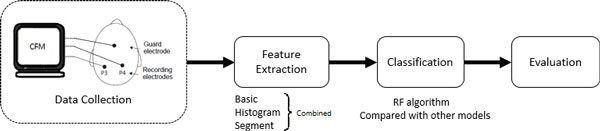
**The block diagram of the RF-based classification system**.

The aEEG classification process is described in details in this section, including data description, the algorithm of random forest, feature extraction, classification and evaluation. Figure 2 gives the block diagram of the RF-based classification system.

### Data description

282 aEEG signals were acquired from Shanghai Children's Hospital of Fudan University, using the Olympic CFM 6000 (Olympic Medical Inc, Seattle, WA). Raw EEG signals were recorded through a pair of biparietal electrodes, and were then filtered, rectified, smoothed and selectively amplified to get aEEG. The positions of the recording electrodes were equivalent to the P3 and P4 electrode positions of the international 10-20 system. The aEEG samples with impedance greater than 10kΩ were discarded. The 282 cases include 209 normal cases and 73 abnormal ones, and the duration of each recording was 3 hours. All the aEEG tracings were interpreted to normal or abnormal ones by experienced clinicians independently.

### Random forest model description

Random forest (RF) developed by Leo Breiman in 2001 has been proved to be a powerful approach with excellent performance in classification tasks [15][16]. Introducing both bagging and random variable selection for tree building, RF utilizes an ensemble of classification trees, which are built on the bootstrap sample of the data. At each split, variable candidate set is randomly selected from the whole variable set. Randomness is injected by growing each tree on different random subsamples and determining splitter partly at random. Each tree is grown fully to obtain a low-bias. Both bagging and random variable selection assure the low correlation for individual trees. Through the averaging over a large ensemble of low-bias, high-variance but low correlation trees, the Algorithm 1 yields an ensemble forest [15].

In this paper, several algorithmic issues were examined, including parameter optimization and imbalanced dataset processing.

**Algorithm 1 **Algorithm of Random Forest

Input:

*T *: Training set $\left({\vec{x}}_{1},y_{1}\right),\left({\vec{x}}_{2},y_{1}\right),\ldots ,\left({\vec{x}}_{n},y_{n}\right)$;

*N_tree_*: the number of trees to be built;

*M_try _*: the number of variables chosen for splitting at each node;

Training:

**for **each *b *= 1 : *N_tree _***do**

 Draw a bootstrap sample *X_b _*from the given training set *T*.

 At each node of tree *tr_b_*, select *M_try _*variables randomly and determine the best split among these *M_try _*variables.

 Construct an unpruned tree *tr_b _*using the above bootstrapped samples.

end for

Classification:

Classify by majority vote among the *N *trees.

Compute

$\begin{array}{c} f_{avg}\left(X\right):=\left(p_{1}\left(X\right),\ldots ,p_{k}\left(X\right)\right):=\frac{1}{N}\overset{N}{\underset{b=1}{\sum }}f_{i}\left(X\right)\\ \;\;\;\;\;\;\;\;f_{RF}\left(X\right):=argmax_{k}\left\{p_{1}\left(X\right),\ldots ,p_{k}\left(X\right)\right\} \end{array}$

#### Parameter optimization

In order to achieve desired performance, two important parameters need to be optimized in the RF algorithm. One is the number of input variables *M_try _*tried at each split, and the other is the number of trees to grow (*N_tree_*) for each forest. *M_try _*considered at each split is a real parameter in the sense that its optimal value depends on the data. The default value (the square root of the number of input variables) is often a good choice for *M_try _*[17]. Generally speaking, the number of trees *N_tree _*in the forest should increase with the number of candidate predictors *M_try _*, so that each predictor has enough opportunities to be selected. To get an appropriate value of *N_tree_*, we can try several increasing values and select the value when the prediction error stabilizes.

#### Imbalanced datasets processing

In our dataset, the number of abnormal data is much smaller than that of normal data. Most machine learning algorithms will perform poorly on the minority class because of the imbalance in the class distribution, and RF is no exception. As the cost of misclassifying of the minority abnormal class is much higher than the cost of other misclassifications, the imbalanced dataset problem is one of the important issues we need to consider to insure a satisfying result.

In this paper, we attempt to make the classifier more robust to the problem of class imbalance by using class weights. A heavier penalty is given when the RF misclassifies the minority class because the classifier tends to be biased towards the majority class [18]. Each class is set a weight, with the minority class given a larger one. Class weights are applied in two places. The first one is in the tree building procedure, where class weights are used to weight the Gini criterion for split point finding. The second one lies in the prediction procedure to produce a "weighted majority vote" by each terminal node. In such a weighted RF model, the final prediction is determined by aggregating the weighted vote from each individual tree. As essential tuning parameters to achieve desired performance, the class weights can be selected through the out-of-bag estimate of the accuracy of RF model [19].

### Feature extraction

Three kinds of features were extracted to characterize the aEEG signals, including basic features, the histogram features from the signal as a whole, and the segment features got from segmented aEEG recordings.

#### Basic Features

Basic features were extracted from the initial 3-hour-length aEEG signal, including minimum amplitude, maximum amplitude, mean value of amplitude and percentage of the lower margin values under 5*µV *. For a 3-hour-length recording, we can get four features.

#### Histogram features

According to the clinical diagnosis criteria, the distribution of aEEG amplitude means a lot for interpretation of the signal [13][20][21][22]. In this work, a histogram of amplitude was calculated to reveal the distribution of aEEG amplitudes.

**Figure 3:**
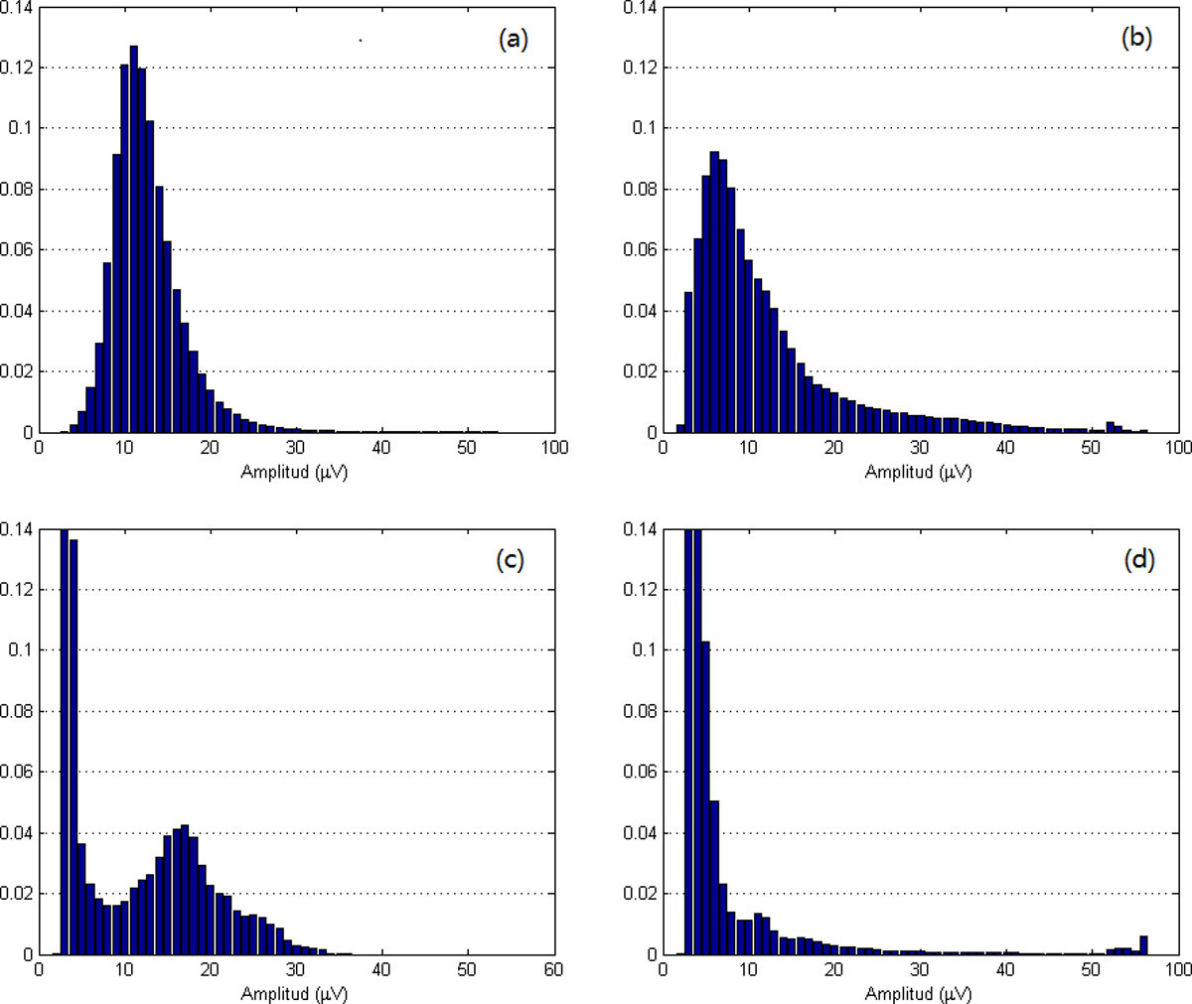
**Amplitude histograms of normal and abnormal aEEG signals: (a) normal; (b)(c)(d) abnormal**.

As aEEG classification is more sensitive to lower amplitude than the higher ones, in our experiments, 1*µV *was used as the width of interval in lower amplitude areas (≤ 50*µV *) and 10*µV *as the width of interval in higher amplitude areas (> 50*µV *). Thus we can get from one 3-hour-length aEEG recording 55 features carrying the histogram information. Four different histogram of normal and abnormal aEEG signals are illustrated in Figure 3. We can observe that, the histogram of normal aEEG looks like the normal distribution (shown in (a)), when those of abnormal aEEG signals are more irregular (shown in (b)-(d)).

#### Segment features

**Figure 4:**
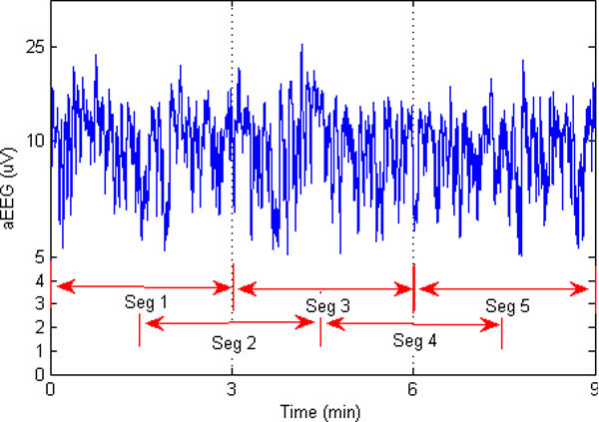
**Data segmentation scheme with overlapped window**. Here a 1.5-min-window overlapping is used, operating on 3 min segments of data.

**Figure 5:**
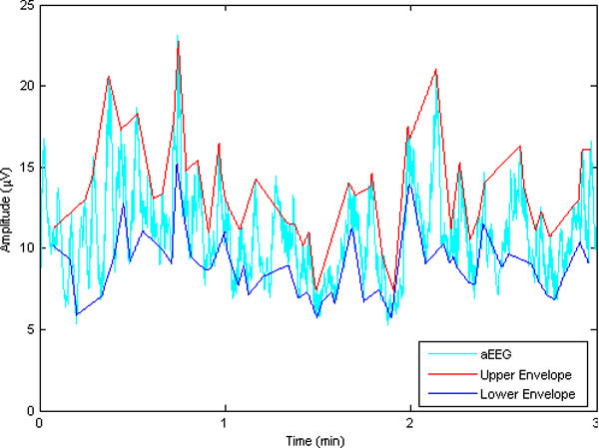
**Data Envelope of aEEG**. The upper and lower boundaries are derived from the mean value of upper and lower envelopes of the segmented aEEG.

To capture subtle difference between normal and abnormal aEEG, the initial 3-hour-length aEEG signals were also segmented into a series of segmentation, and then features were extracted from the segmented series. The overlapped windowing data segmentation scheme was used to catch more detailed information, which is demonstrated in Figure 4. Here the length of the segment window was set to 3 min and an overlap of 1.5 min was used. Experiments show that such a selection works well in most scenarios with reasonable computing capacity. Four features were examined for each segment, including the upper boundary, the lower boundary, the mean value and approximate entropy (ApEn). The mean value is the mean of the amplitudes of the segmentation. The upper and lower boundaries are derived from the envelope of the segmented aEEG. The second order envelope was calculated for each segment. Averaging the upper and lower envelop, we got the upper boundary and the lower boundary of one segment (illustrated in Figure 5).

Approximate entropy [23][24] is a good description of aEEG signals helpful in detecting brain disorders of the newborn. ApEn can be briefly described as follow: Given a time-series *u*(1)*, u*(2)*, . . . , u*(*N *), a vector in *m *dimensions is defined as $X_{i}^{m}=\left[u\left(i\right),u\left(i+1\right),\ldots ,u\left(i+m+1\right)\right]$, where *m *is the length of comparing window. And a sequence of vectors $X_{1}^{m},X_{2}^{m},,\ldots X_{N-m+1}^{m}$ can be constructed. For each *i*, 1 *≤ i ≤ N − m *+ 1, let $C_{r}^{m}\left(r\right)$ be (*n − m *+ 1)*−*1 times the number of vectors $X_{j}^{m}$ within tolerance *r *of $X_{i}^{m}$. Then we can define Φ*_m_*(*r*) as the following formula from $C_{r}^{m}\left(r\right)$:

(1) $${\text{Φ}}_{m}\left(r\right)=\frac{\sum_{i=1}^{N-m+1}lnC_{i}^{m}\left(r\right)}{\left(N-m+1\right)},$$

where *ln *is the natural logarithm. Given a fixed positive integer *m *and a positive real number *r*, ApEn(m,r) is defined by

(2) $$ApEn\left(m,r\right)=\underset{N\to \infty }{\text{lim}}\left[{\text{Φ}}^{m}\left(r\right)-{\text{Φ}}^{m+1}\left(r\right)\right].$$

For a fixed *N *data points, it is defined as

(3) $$ApEn\left(m,r,N\right)={\text{Φ}}^{m}\left(r\right)-{\text{Φ}}^{m+1}\left(r\right).$$

For one 3-hour-length aEEG recording, 80 segments were observed. And for each segment, we can get four features: the upper boundary, the lower boundary, the mean value and ApEn. Thus for one 3-hour-length recording, we can get 320 features. Obviously it is time consuming if all these features are sent into a classifier. To speed up the classification processing, it's wise to reduce the dimension of the feature vector by ignoring those unimportant ones. According to our previous work [23], ApEn with higher or lower values may more likely indicate the abnormality of a signal. So the segment features were firstly sorted in an ascending order according to the values of ApEn, and then only those segments with high and low values of ApEn are selected. Through experiments, we picked up the segment features with the ten top and the five bottom values of ApEn, and thus we got a 60-dimensional feature vector for one 3-hour-length recording.

After the basic features, histogram features and segment features had been got respectively, they were integrated into one combined feature set with 119 features.

### Classification

The weighted RF was applied to classify the 282 aEEG signals based on the feature sets got above. To evaluate the performance of RF on aEEG classification, other widely used classifiers were also tested on the same data sets and the identical feature sets. The compared classification methods include the support vector machine with RBF kernel (SVM-RBF), support vector machine with linear kernel (SVM-Linear) and artificial neural network (ANN). As a reference, we also considered the Maximum Likelihood (ML), Decision Tree using CART (DT), Logistic Regression (LR), Linear Discriminant Analysis (LDA) algorithm, four of the most popular traditional supervised classification methods.

### Performance evaluation

Instead of using cross validation or estimating from a separate testing, an unbiased error can be estimated internally in random forest [25]. Each tree is constructed under a different bootstrap sample. About one-third of the samples are left out of the bootstrap sampling and not used in the construction of the tree, so the left out samples can be put into the tree as test samples. At the end of the procedure, we took the number to be the class that got most of the votes every time. By calculating the proportion of misclassified samples over all cases, we can get the OOB error estimation, which has been proved to be an unbiased error estimation method for random forest [26].

Specificity, sensitivity and *F*_1_-score were applied to evaluate the performance of the classifiers. The specificity is defined as the percentage of the number of true negatives over the sum of the number of true negatives and that of false positives. The sensitivity refers to the percentage of the number of true positives over the sum of the number of true positives and that of false negatives. The *F*_1_-score can be interpreted as a harmonic compromise of precision and recall, which reaches its best value at 1 and worst score at 0 [27].

## Results

We conducted a series of experiments on 282-subject dataset to achieve an optimum configuration of the RF-based classifier. The experimental study can be divided into three parts. The first set of experiments examined the effects of parameters *N_tree_, M_try _*and evaluated the candidate feature sets. In the second set of experiments, we dealt with the problem of imbalanced datasets. And the third set of experiments compared the performance of RF-based classifier with those of other classifiers.

### Parameters tuning and feature evaluation

As there are two relevant parameters to be optimized in RF algorithm, we have to try out one of the parameter with the other one supposed to be given.

**Figure 6:**
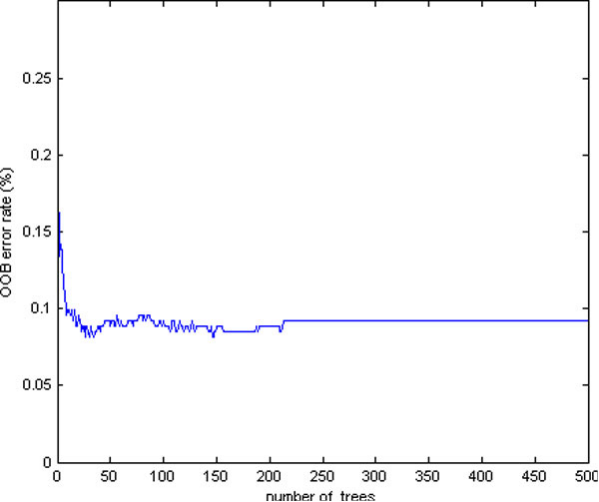
**Random Forest parameter selection**. The average Out of Bag (OOB) error with default *M_try _*for different forest sizes. The size should be greater than 220 with a stable OOB error of 0.083.

**Figure 7:**
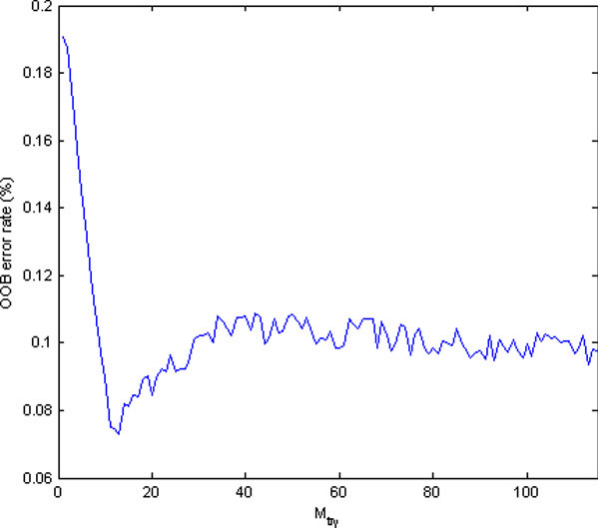
**Random Forest parameter selection**. The average OOB error for different value of *M_try _*with *N_tree _*= 1000. The optimal value of *M_try _*with the lowest OOB error occurs near the default parameter of *M_try _*= 11.

To select an appropriate value of *N_tree_*, we initially built forest with a default parameter of $M_{try}=\sqrt{119}\approx 11$, and then the average Out of Bag (OOB) error was examined in different forest sizes *N_tree_*. Figure 6 illustrates the change of OOB error with increasing forest size *N_tree _*under default *M_try _*. It can be observed that the OOB error tends to be stable at 0.083 when *N_tree _*increases to about 220. As RF works better with greater *N_tree_*, we selected 1000 as the forest size in our further experiments. After *N_tree _*had been set, forests were built with varying values of *M_try _*from 1 to 119. Figure 7 shows the OOB error with different *M_try _*values. The optimal value of *M_try _*with the smallest OOB error occurs near the default parameter of *M_try _*= 11, which indicates that the optimal *M_try _*really occurs near the default value. Based on the experiments, a final random forest model was generated using the parameters *N_tree _*= 1000 and *M_try _*= 11.

To assess the feature sets, we worked out the significance of feature as following: In every tree grown in the forest, first write down the OOB cases and count the number of votes for the correct class, and then permute the values of feature *x_i _*in the OOB cases randomly and put these cases into the tree classifier. For each tree *t*, subtract the number of correct votes in the feature-*x_i_*-permuted OOB data from that of correct votes in the unpermuted OOB data:

(4) $$FI^{t}\left(x_{i}\right)=\frac{\sum_{i\in {\bar{B}}^{t}}I\left(y_{i}={\overset{\text{\textasciicircum{}}}{y}}_{i}^{t}\right)}{\left|B^{t}\right|}-\frac{\sum_{i\in {\bar{B}}^{t}}I\left(y_{i}={\overset{\text{\textasciicircum{}}}{y}}_{i,\pi_{j}}^{t}\right)}{\left|B^{t}\right|}$$

(5) $${\overset{\text{\textasciicircum{}}}{y}}_{i}^{t}=f^{t}\left(x_{i}\right)\;\;:\text{predicted}\;\text{class}\;\text{before}\;\text{permuting};$$

(6) $${\overset{\text{\textasciicircum{}}}{y}}_{i,\pi_{j}}^{t}=f^{t}\left(x_{i,\pi_{j}}\right)\;\;:\text{predicted}\;\text{class}\;\text{after}\;\text{permuting}\;,\pi_{i};$$

The average of *FI^t^*(*x_i_*) over all trees in the forest is the raw significance score for feature *x_i _*as in Equation 7:

(7) $$I_{x_{i}}=\frac{\sum_{t=1}^{N_{tree}}FI^{t}\left(x_{i}\right)}{N_{tree}}.$$

**Figure 8:**
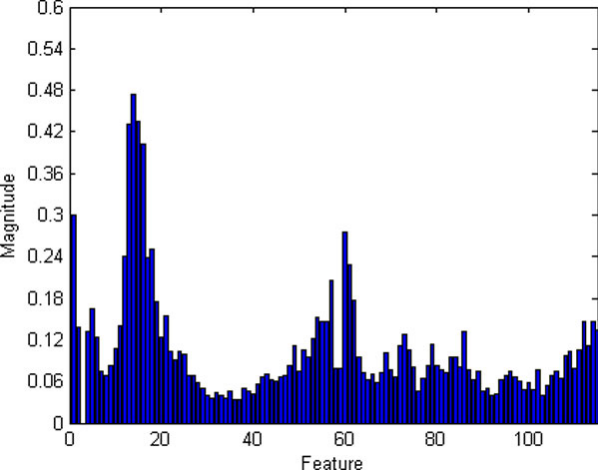
**Significances of Features**. Features are arranged on x-axis by their serial number in the feature vector.

**Figure 9:**
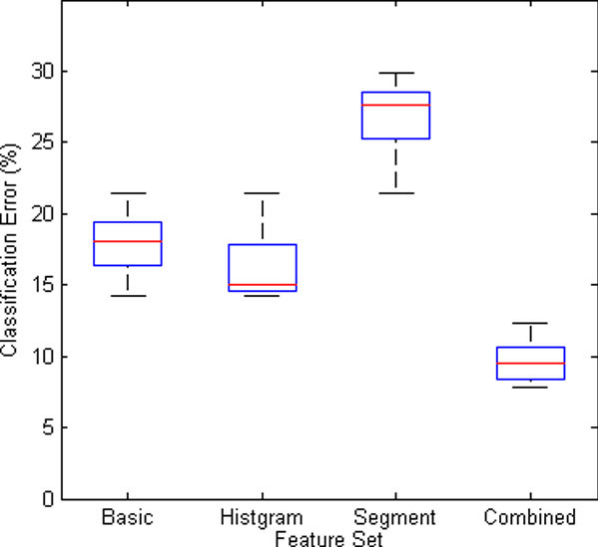
**The classification error of the four kinds of feature sets**.

The significances of all the four kinds of features are shown in Figure 8. Most features' significances in the original feature set are greater than 0.05. To find the most valuable features, the basic, the histogram and the segment features, as well as the combined features were sent to a RF-based classifier respectively. The classification results based on the four kinds of feature sets are depicted in Figure 9. It is obvious that the combined feature set works the best among the four, with a minimum median and narrowest variation of classification error.

### Imbalanced datasets processing

For weighted random forest, we tuned the class weight for final prediction: when we raise the minority class weight, the cost of misclassification of the minority class goes up, thus we can get a higher true positive rate and a lower true negative rate. To balance the sensitivity and specificity, the geometric mean (G-mean) was applied. A distinctive property of the G-mean measure is that it is independent of the distribution of classes. It will reach its best value when sensitivity and specificity are performed well at same time. G-mean can be calculated by Equation 8:

(8) $$g=\sqrt{Sensitivity*Specificity}.$$

**Figure 10:**
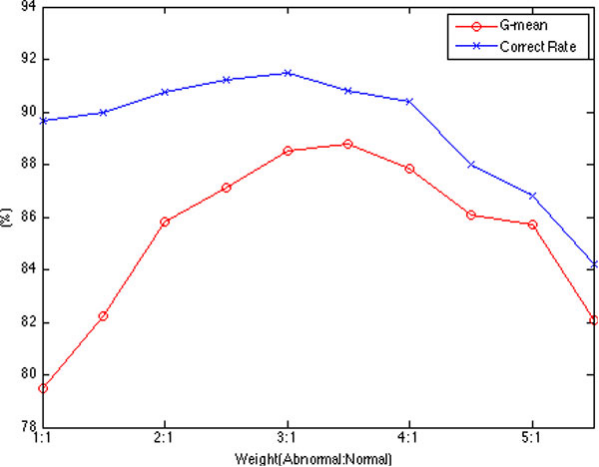
**The correct rate and G-mean of the weighted random forest with different weights**.

Figure 10 gives the correct rate and G-mean of the weighted random forest with different weights. The results show that the model works the best when the weight of the abnormal and normal class is assigned to 3:1.

### Classification

To appraise the performance of the RF-based classifier, some widely used classifiers, including the SVM-RBF, SVM-Linear, ANN, DT, LR, ML, and LDA, were also applied to classify the identical data based on the identical feature sets. OOB method and 10-fold cross validation method were utilized to evaluate the prediction ability of RF and those of other classifiers respectively. Further more, we compared the performance of RF build on different feature sets. Based on previous analysis, we can select part of features with high significance to build our model for acceptable accuracy and efficiency.

Table 1 describes the results of classification. In this table, RF with the optimal parameters outperforms all of the other classifiers. The RF method gets the highest correct rate, sensitivity, specificity, as well as *F*_1_-score among the seven classifiers. Table 2 shows the performances of RF model trained with different feature sets. During the model training, we selected the top n% (from 10% to 100%) features based on its significance score to build our model.

**Table 1:** Performance comparison for different classifiers.

Method	Correct Rate	Sensitivity	Specificity	*F*_1_-score
RF	**92.52**%	93.78%	87.50%	**95.26**%
SVM RBF	89.67%	91.56%	82.14%	93.52%
SVM Linear	86.12%	89.19%	74.58%	91.03%
ANN	91.10%	92.00%	87.50%	94.31%
DT	87.90%	85.83%	61.76%	71.84%
LR	85.40%	88.31%	68.29%	77.03%
ML	75.44%	78.67%	62.50%	83.69%
LDA	72.95%	73.33%	71.43%	81.28%

**Table 2:** Performance comparison for feature set under different sizes.

Selected feature set size(%)	Correct Rate	Sensitivity	Specificity	*F*_1_-score
100	92.17%	93.33%	87.50%	95.07%
80	91.81%	92.89%	85.71%	94.78%
60	90.62%	90.19%	87.58%	93.03%
40	88.67%	91.11%	83.93%	91.37%
20	83.33%	96.33%	32.14%	89.59%
10	80.91%	95.56%	32.29%	88.03%

## Conclusions

In this paper, we proposed a RF-based method for aEEG classification and defined a combined feature set. Basic features, statistical features and segment features were extracted from the whole signal as well as from signal segmentations. The combined feature set consisting of the three kinds of features was then sent to the RF classifier. The significance of feature, the data segmentation, parameter optimization of RF algorithm, and the problem of imbalanced datasets were examined. Experiments were also conducted to evaluate the performance of RF on aEEG classification, compared with several other widely used classifiers. Results show that, outperforming other widely used classifiers examined here, random forest with the combined feature set is efficient and can help better detect the brain disorders in newborns.

## Competing interests

The authors declare that they have no competing interests.

## Authors' contributions

The work presented here was carried out in collaboration among all the authors. WC, GTC and YW designed the methods and experiments. WC and YW carried out the experiments and wrote the manuscript. GQC, QFG recorded and analyzed the data, interpreted the results and helped to write the manuscript. All the authors approved the manuscript.

## Declarations

Publication of this article has been funded by the National Natural Science Foundation of China (Grant No. 81101119), Natural Science Foundation of China (Grant No. 61340036), the Open Project of Software/Hardware Co-design Engineering Research Center MoE, and National Key Basic Research Program (Grant No. 2011CB707104).

This article has been published as part of *BioMedical Engineering OnLine *Volume 13 Supplement 2, 2014: Selected articles from the IEEE International Conference on Bioinformatics and Biomedicine (BIBM 2013): BioMedical Engineering OnLine. The full contents of the supplement are available online at http://www.biomedical-engineering-online.com/supplements/13/S2.
